# Othering in the nursing context: A concept analysis

**DOI:** 10.1002/nop2.82

**Published:** 2017-05-07

**Authors:** Mary Lee A. Roberts, Martin Schiavenato

**Affiliations:** ^1^ College of NursingWashington State UniversitySpokaneWAUSA

**Keywords:** diversity, dominant power, dominant‐subordinate relationship, exclusion, inclusion, othering, the other

## Abstract

**Aim:**

‘Othering’ is described as a social process whereby a dominant group or person uses negative attributes to define and subordinate others. Literature suggests othering creates exclusive relationships and puts patients at risk for suboptimal care. A concept analysis delineating the properties of othering was conducted to develop knowledge to support inclusionary practices in nursing.

**Design:**

Rodgers’ Evolutionary Method for concept analysis guided this study.

**Methods:**

The following databases were searched spanning the years 1999–2015: CINAHL, PUBMED, PsychINFO and Google. Search terms included “othering”, “nurse”, “other”, “exclusion” and “patient”.

**Results:**

Twenty‐eight papers were analyzed whereby definitions, related concepts and othering attributes were identified. Findings support that othering in nursing is a sequential process with a trajectory aimed at marginalization and exclusion, which in turn has a negative impact on patient care and professional relationships. Implications are discussed in terms of deriving practical solutions to disrupt othering. We conclude with a conceptual foundation designed to support inclusionary strategies in nursing.

## 1 Introduction

In her illustrative and appropriately titled work, *The Second Sex*, Simone de Beauvoir provides a formative analysis of othering by specifying the ways men have consistently exerted their personal power to define women as “The Other” (De Beauvoir, 1989). Herein, de Beauvoir explains how men have often initiated a process of‐ othering by differentiating women according to a dominant male standard: “She is defined and differentiated with reference to man and not he with reference to her; she is the incidental, the inessential as opposed to the essential. He is the Subject, he is the Absolute – she is the Other” (De Beauvoir, 1989, p. xxii). In feminist literature this interpersonal process of generating of “the other”, otherwise identified simply as othering, has been used to describe a sequence of events, whereby a person or group is differentiated from a dominant group in accordance with a chauvinistic social standard (Anzaldua, 1987). On the whole, nursing research has supported this premise, while indicating that difference is a foundational attribute to the othering process (Canales, 2010). In a similar way, social theorists have described othering as a process by which interpersonal differentiation generates a distinct form of social exclusion and subordination (Bourdieu, 1984, 1998; Dervin, 2012).

### 1.1 Background

In the early 2000s, nursing researchers began using these and similar descriptions to work at framing othering as a mostly negative and exclusionary process that can often times be based on racial and/or ethnic biases (Canales, 2000, 2010). For example, it was in these explorations into othering in nursing practice where the othering concept emerged as a useful descriptor of adverse nurse‐patient encounters, especially in cases where patients were differentiated into racial and ethnic categories and treated in a subordinating way (Browne, 2007; Johnson et al., 2004). And even though these studies provided ample patient testimony describing the negative and exclusionary environments associated with nurse‐patient othering, no elemental definition aimed at a specific formulation for how othering operates as an inter‐personal social process was proposed, either for nurse‐patient, nurse‐nurse, or inter‐professional relationships.

More recently, research has explored how nurse‐to‐nurse gender‐based differentiation has an impact on the experiences of both male nurses and male nursing students, particularly in settings where the male nursing identity is marginalized by a dominantly female‐centric nursing workforce (MacWilliams, Schmidt, & Bleich, 2013). Additional research has worked to align conceptualizations of othering in relation to inequities inherent to inter‐professional relationships, such as the nurse‐physician relationship (Weeks, 2005). And although these studies focus on a range of distinct populations, what remains consistent across nursing literature is the premise that othering can potentially develop into exclusionary patterns of behaviour; the outcome of which is a perpetuation and reinforcement of already existing healthcare, educational and workplace inequities based on gender, race, ethnicity and other categorical social constructs (Ellis, Meeker, & Hyde, 2006; Vasas, 2005; Weeks, 2005).

In exploring options aimed at reducing the impact of exclusionary social processes on patient care, a series of reports emerging from the Robert Wood Johnson Foundation (RWJF) Initiative on the Future of Nursing suggest that gender diversity and inter‐ethnic inclusion in the nursing workforce can lead to improvements in patient care (Institute of Medicine, 2011; Robert Wood Johnson Foundation, 2012). And indeed, several of these RWJF reports indicate that a nurse‐patient inclusionary understanding of gender and ethnic differences promotes better nurse‐patient communication and healthcare outcomes, as some patients are more receptive to healthcare providers from similar backgrounds and gender (Ayoola, 2013; Lecher, 2014). Hence, with men comprising less than 10% of the current nursing workforce and with only 19% of registered nurses being from ethnic minority backgrounds (United States Health Resources Services Administration, 2014), findings from these RWJF reports suggest that the nursing profession should confront two direct challenges: (i) Design and implement initiatives aimed at supporting a more gender and racially diverse nursing workforce; and (ii) Identify and disrupt exclusionary social processes (e.g. othering), which counteract efforts aimed at advancing diversity and inclusion in nursing practice and across the nursing workforce (Institute of Medicine, 2011; Lecher, 2014; Robert Wood Johnson Foundation, 2012).

To address these challenges, this concept analysis aims to define othering by analyzing its elemental properties in: nurse‐patient, nurse‐nurse and inter‐professional relationships, using the Rodgers’ Evolutionary Method for concept analysis (Rodgers & Knafl, 2000). In following a precedence set forth in previous investigations into othering (Canales, 2010) and to sharpen our focus on how othering emerges in interpersonal contexts as an operative exclusionary process, this concept analysis will incorporate writings from feminist and social theorists who have specifically addressed interpersonal exclusion and differentiation. The fundamental objectives of this concept analysis are: (i) Explore the potential for concept‐based findings to inform efforts aimed at disrupting the othering process in nursing; (ii) Provide information to support gender and ethnic diversity in the nursing workforce; and (iii) Investigate formulations for inclusionary practices designed to improve patient care.

## 2 Methods

The Rodgers’ Evolutionary Method, an approach specifically developed to analyze an evolving concept based on an interdisciplinary inquiry, served as a procedural guide for this concept analysis (Rodgers & Knafl, 2000). Our use of this methodological approach was divided into three phases following Tofthagen and Fagerstrom (2010): (i) The Initial Phase: concept and context are identified and literature is reviewed to obtain data for analysis; (ii) Findings: definitions, related concepts, antecedents, exemplar and attributes are identified; and (iii) Discussion: consequences, conclusion and implications for further research. Ethical approval was not required for this study.

### 2.1 The initial phase

#### 2.1.1 Concept and context are identified

In its most basic conceptualization, othering serves to empower individuals by motivating them to notice comparable differences in others and the particular ways these “others” do not adhere to a dominant social standard (Gillespie, 2007). A nurse exerting his/her power to reinforce interpersonal differences based on a dominant social standard—often in terms of race, ethnicity and/or gender—is a recurring example of othering in literature. In this context, nursing literature has described othering as a process that reinforces a dominant nursing standard and generates exclusionary, albeit negative relationships amongst healthcare professionals and between patients and nurses (Browne, 2007; Canales, 2010; Kada, 2010).

#### 2.1.2 Literature review and data for analysis

The following databases were searched: CINAHL, PUBMED, PsychINFO and Google Scholar. Keyword and title searches for the terms “othering”, “nurse”, “other”, “exclusion” and “patient” were carried out for the years 1999–2015. All documents were limited to the English language. These searches yielded a group of 308 sources, which ultimately was reduced to 152 unique documents after accounting for the overlap between databases. Search results were further limited by their specificity to the stated population and the objectives indicated for this analysis, which resulted in a definitive group of 28 sources that met the limits of these criteria.

## 3 Findings

### 3.1 Definitions

Nursing researchers have regularly used and adopted social theoretical conceptual frameworks to formulate descriptions of othering, where social theorists have explained and described othering and related exclusionary relationships in terms of a sequential process culminating in inter‐relational differentiation and subordination (Bourdieu, 1984; Burgess & Park, 1969; Said, 1993). This process can typically commence with a given dominant person or group recognizing the existence of an accepted “normal” social standard for behaviour, which in turn facilitates the operability of a social environment defined by this dominant person or group. Hence, when this dominant‐defined social environment is challenged and/or stressed by individuals who do not conform to this normal social standard, the dominant person or group responds by identifying and differentiating, that is, othering these non‐conforming individuals, who are then relegated to the status of “the other”. In creating difference through othering, potentially the dominant person or group is empowered while the non‐conforming “others” are consigned to a substandard status, which in theory would restore the dominant‐defined social environment to its former prominent status (Bourdieu, 1998; Burgess & Park, 1969; Canales, 2000).

Nursing literature has appended to this conceptualization of othering in several fundamental ways. For example, MacCallum (2002) has accentuated the dual importance of exclusion and identity in othering, saying that a group of nurses or patients “is not necessarily defined by those who are in it but by those who are excluded from it”(p. 87). And where social theorists have suggested that the basic operations and interpersonal processes related to othering are a mostly unconscious process (Bourdieu, 1998), nursing literature has recognized both conscious and unconscious formulations of othering. Hence, Canales (2000) has suggested that othering can at times be manifested as both a conscious and intentional process, while Mee (2012) and Browne (Browne, 2007) suggest that othering is frequently an unconscious, albeit unintentional practice in the nursing profession.

### 3.2 Related concepts

In accordance with its definitional characteristics, othering has principally been associated with exclusionary concepts and social practices. However, with further study and investigation, we find that the othering concept's association with exclusionary social practices has merely served as a point of reference in nursing; as it is evident that several nursing researchers have gone on to explore alternative othering‐related concepts and proceeded to reformulate othering into an inclusionary, rather than exclusionary, social process (Canales, 2010). For this reason, we present concepts related to othering that are characterized by exclusionary practices, as well as those concepts that appear as inclusionary re‐formulizations of othering and work to construct inclusionary relationships in nursing practice.

### 3.3 Exclusionary concepts

Nursing literature has consistently associated othering with these related concepts: marginalization, stereotyping and racialization (Browne, 2007; Canales, 2010; Evans, 2002; Tang & Browne, 2008; Vasas, 2005). In a nursing research concept analysis of marginalization, Vasas (2005) has suggested that marginalization can be described as a distinct process that enforces the dominant/subordinate social differences created by othering. In following this definition, marginalization can therefore be understood as an outcome of othering. For example, nursing literature refers to patients subordinated by a dominant bio‐medical othering process as subsequently being marginalized to an excluded status in the healthcare system (Meleis & Im, 1999). In a making a similar association with othering, nursing research has conceptualized stereotyping as a tool used by a dominant nursing group to classify patients or colleagues who do not conform to a normal social standard (Drevdahl, Canales, & Dorcy, 2008; Tang & Browne, 2008). In this sense, stereotyping is defined as a formulaic oversimplification of an individual or group identity and may be considered a co‐process of othering that facilitates classification and exclusive social groupings (Browne, 2007). Racialization, in turn, has been considered a subcategory of stereotyping based on the concept of race (Tang & Browne, 2008).

Significantly, numerous sources in nursing literature have described marginalization, stereotyping and racialization as predominantly exclusionary processes that are related in some way or form to othering (Browne, 2007; Canales, 2010; Tang & Browne, 2008; Vasas, 2005). As will be further elaborated on below, this fundamental and powerful connection between exclusion and othering, as exemplified in instances of exclusionary othering in nursing practice, has had a considerable impact on the quality of patient care.

### 3.4 Inclusionary concepts

#### 3.4.1 Inclusionary Othering

In contrast to the more ubiquitous exclusion‐based characterizations of the othering process, Canales (2010) has documented multiple situations where marginalized nurses, as well as researchers and scholars, have used the inter‐relational capacities of othering in an antithetical way and reformulated exclusionary othering into an inclusionary social process. In a ten‐year retrospective analysis of othering (spanning 2000–2010), Canales (2010) presents a survey of the othering concept in nursing literature, including both exclusionary and inclusionary conceptualizations. Of particular note herein is Canales’ original conceptualization of Inclusionary othering, which is offered as a positive countermeasure to the inherently negative exclusionary form of othering. As was first documented in her research circa 2000, Canales (2000) has described Inclusionary othering as “a process that strives to connect through difference” (p. 28). In these terms, Inclusionary othering uses the recognition of difference for alliance building, rather than as a mechanism for reinforcing marginalization and exclusion.

#### 3.4.2 Role‐taking and world‐travelling

In her early investigations into inclusionary social practices, Canales (2000) initially situated her formulation for Inclusionary othering in the conceptual context of role‐taking and world‐travelling; two related concepts originating in social theory and feminist literature that serve as constructs for building inclusionary relationships. Similar to Canales’ ‘inclusionary othering’, nursing researchers have further defined and subsequently used themes and variations on the role‐taking and world‐travelling concepts to develop nursing interventions aimed at countering the negative effects exclusionary othering. For example, Burbank and Martins (2010) have worked to define a relational mechanism for role‐taking, or “taking on the role of the other”, where a recognition for the positive aspects of social difference becomes the means by which coalitions are built based on an interpersonal understanding between nurses, patients and colleagues. In applying the role‐taking concept to nursing education, Phillips and Peterson (2005) have constructed a comparable formulation for “taking on the role of the other”, which they have used to develop a nursing education curriculum designed to enhance a nursing student's awareness for the situational circumstances of marginalized patients.

In a similar conceptualization to role‐taking, feminist scholar and writer María Lugones (1987) has presented world‐travelling as way to build relationships based on an understanding of interpersonal difference. Herein world‐travelling is conceptualized as an extension of oneself by “travelling” to the world of “the other”. As Lugones (1987) explains, “Only when we have travelled to each other's ‘worlds’ are we fully subject to each other” (p. 17). Bunkers (2003) has proposed two nursing practice‐based manifestations for world‐travelling, which are formulated to connect with patients and/or colleagues who may be at risk for, or potentially be socially situated as “the other”. In this context, Bunkers (2003) sets forth two objectives: (i) Creating a hospitable patient care environment; and (ii) “attending to others with true presence,” as ways to realize world‐travelling and “push the edges of the boundaries of lived experiences…[by creating] possibilities for connecting with *the stranger* [‘the other’]” (pp. 307‐308).

Lastly, it is important to note that these inclusionary processes (inclusionary othering, role‐taking, world‐travelling) often times arise from and emerge in the negative nature and consequences of the exclusionary form of othering. That is, these inclusionary responses to exclusionary othering may be considered as inherently positive reformulations of the othering process, inasmuch as they work toward manifesting positive outcomes from potentially negative circumstances (Canales, 2010).

### 3.5 Antecedents and exemplar

To develop distinction for an evolving concept, Rodgers’ Evolutionary Method prescribes a delineation of antecedents and an identified exemplar. Antecedents are pre‐existing actions or causes that are consistently associated with a concept. The exemplar serves to illustrate how a concept operates in the nursing practice context (Rodgers & Knafl, 2000).

Nursing literature has consistently presented two essential antecedents of othering: the normal social standard and dominant power (Canales, 2010; Johnson et al., 2004; MacCallum, 2002; Weeks, 2005). In addition, it is noteworthy that literature has often characterized sources of dominant power, as well as normal social standards in such a way that they appear to instigate and activate the othering process (Canales, 2000; Johnson et al., 2004). In this sense, the normal social standard and dominant power antecedents co‐operate by working to identify and create difference with non‐conforming individuals, which in turn instigates the othering process and subordinates these individual(s) to a substandard status. The following is an exemplar from nursing literature showing how the normal social standard and dominant power antecedents can become operational in a nursing care setting and generate multiple instances of othering along with a series of potentially negative outcomes.

### 3.6 The exemplar(s)

The exemplar chosen for this concept analysis is in fact a compilation of exemplary cases of othering based on Browne's (2007) ethnographic study: *Clinical encounters between nurses and First Nations women in a Western Canadian hospital*, which describes the experiences of Canadian nurses and First Nations (Aboriginal or Indigenous) women in a healthcare setting. Herein Browne describes a situation where nurses use a dominant discourse based on assumptive cultural differences to reinforce several normal social standards. In this context, Browne shows how nurses have supported dominant social standards while deriving personal power from reinforcing their assumptions that First Nations patients, in numerous and sometimes subtle ways, do not conform to the socio‐cultural standards set forth by the dominant Canadian cultural power. Before proceeding, it is important to recognize that Browne's findings aim to show how, “nurses (and other providers) are *not* intentionally [emphasis added] engaging in othering practices” (Browne, 2007, p. 2724). Still, even in this framework, Browne's findings indicate that the normal social standard and dominant power antecedents remain to be effectual and potentially insidious components of the othering process.

#### 3.6.1 Othering exemplars

In multiple observational encounters/sessions where researchers “shadowed” nurses, Browne (2007) has captured recurring sequences of nurses unintentionally using a socio‐cultural standard, along with their (the nurses’) dominant power status, to initially identify and differentiate First Nations patients and then marginalize these patients to a substandard status. For example, these nurses were quoted as saying: “Some of them [First Nations patients] don't like us at all. You know it's been inbred in them from a very young age. They resent us” (Browne, 2007, p. 2171). While other nurses were found to assume that First Nations patients could potentially be “unclean or…vectors for infections” (p. 2171). It is noteworthy that many nurses in this study provided commentary explaining how First Nations patients are “different,” (p. 2171) especially in terms of communication styles, but also in seemingly analytical terms, where one nurse is quoted as saying, “What I find is that some patients, the Natives in particular, have a propensity to like narcotics” (p. 2174).

Browne (2007) also describes how nurses have used their collective power to identify and differentiate the “angry” (p. 2171) First Nations women and strategize ways to reduce contact with these patients. Herein Browne (2007) comments that nurses were: “Guarding against how the other [First Nations patient] might react [which] contributed to an observable form of social distancing that sometimes occurred between the nurses and some of the First Nations women they cared for” (p. 2171). Hence, by exerting their dominant power to enforce a normal social standard, these nurses effectively situated the First Nations patients into the role of the other, thus providing several exemplary and illustrative cases of othering in nursing practice.

Browne (2007) concludes her documentation of this study by noting the clinical outcomes of the othering process:

The consequences in terms of clinical practice are not insignificant. Patterns of social distancing, shaped by processes of othering, limit possibilities for therapeutic engagement particularly in relation to patients’ psychosocial, emotion or material needs (p. 2175).

### 3.7 Attributes identified

Attribute identification provides a coherent basis for making a concept manifest and distinguishable (Rodgers & Knafl, 2000). Nursing literature has consistently listed the following attributes as part of a conceptualization of othering: the dominant‐subordinate relationship, difference, identity and exclusion (Canales, 2000, 2010; Drevdahl et al., 2008; Johnson et al., 2004; MacCallum, 2002). The following describes the tangible ways these attributes operate as definitional qualities of othering and function as conceptual support mechanisms for the othering process in nursing practice.

### 3.8 Dominant‐subordinate relationship, difference and identity

Findings from nursing literature indicate that the dominant‐subordinate relationship, difference and identity attributes function as interconnected collaborative operators aimed at manifesting an occurrence of othering. Hence, each attribute is formulated in a causal relationship with the other two, collectively working to generate a coherent course or trajectory. In this way, othering is ostensibly identified as a process of differentiation and self‐identity reinforcement, which is sustained and supported by the dominant‐subordinate relationship (Canales, 2000; Drevdahl et al., 2008; Gillespie, 2007; MacCallum, 2002).

Nursing researchers have described the causal relationship between the differentiation and subordination of “the other” as a self‐perpetuating process used by a dominant force to consolidate and increase its identity and power. For example, Weeks (2004, 2005) has incorporated this premise into two studies of dominant‐subordinate/physician‐nurse relationships in perioperative and acute care settings. These studies contend that to maintain a powerful identity, the dominant physician entity frequently initiates the othering process to subordinate nurses in practice situations (Weeks, 2004, 2005). In turn, this initiation of the othering process has the potential to generate an impetus for nurses to assert their power as a dominant identity over their subordinates, who may be patients and/or other nursing colleagues. Feminist theorists postulate that this initial assertion of a dominant power (such as that originating with the dominant physician entity) sets in motion a sequential progression, whereby a series of top‐down/dominant‐to‐subordinate processes of differentiation and subordination occur (Jackson, 1999). Canales (2000) offers a perspective on othering that resonates with this premise, which suggests that the othering process is dependent on the necessary attribute of differentiation and has the potential to reinforce a dominant identity through a series of inter‐dependent dominant‐subordinate relationships.

### 3.9 Exclusion

The premise for the dominant‐subordinate inter‐dependent relationship has been a recurring and powerful stimulus for feminist and social theorists to further define the paradoxical identity of the socially excluded “other” (Jackson, 1999). The essence of this paradox lies in the fact that the othering process, while exemplifying the subordinate identity as visibly different, also excludes subordinate individuals from the dominant social context. In this way, the subordinate identity is made visible by differentiation, while simultaneously being made invisible by exclusion (Madrid, 1995). It is in these terms that exclusion emerges as an essential attribute to the othering process.

In a similar way, nursing researchers have explored how males, as a gender minority in the female dominated nursing profession, are at once seen as visibly different, while being excluded from fully participating in and contributing to the advancement of nursing practice (Evans, 2002; O'Lynn & Tranbarger, 2007). In a multi‐national literature review, MacWilliams et al. (2013) present evidence of the ways the dominantly female‐oriented nursing profession and educational academy have excluded a male‐oriented perspective on nursing and effectively reduced the male identity in nursing into a binary categorization of “gay/emasculated” or “he‐man/masculine” stereotypes (MacWilliams et al., 2013, p. 41).

In nursing education, male students claim that this binary categorization is partly due to the lack of male nursing faculty and the scarcity of male professional role models, both of which reinforce the notion that the male presence in the nursing profession should be viewed as different and therefore non‐standard (O'Lynn, 2004). In the midst of this differentiation, male nursing students have experienced the direct and formative impact of being excluded from the female‐dominated nursing profession, which has the potential to undermine their (the male nursing students’) efforts to develop a male‐centric professional identity in preparation for a career in nursing (McLaughlin, Muldoon, & Moutray, 2010; O'Lynn, 2013; O'Lynn & Tranbarger, 2007). This compelling evidence illustrates how the difference and exclusion attributes of othering can work in tandem to have an impact both on individuals and their group identity.

## 4 Discussion

### 4.1 Consequences

It is significant that both parties in the dominant‐subordinate relationship experience a muted capacity for self‐knowledge. For example, a male nursing student gains quantities of knowledge concerning the dominantly female‐centric nursing experience, but possibly at the expense of an equal opportunity to develop a personalized male‐centred identity for nursing practice (O'Lynn & Tranbarger, 2007). On the other hand, the dominantly female‐based paradigms perpetuated in nursing education impede the academy's capacity to evaluate its own curriculum and find ways to learn from and incorporate the male nursing experience (Grady, Stewardson, & Hall, 2008; Kirk, O'Lynn, & Ponton, 2013). Similarly, dominant culture and bio‐medical standards often times work in tandem to discourage nurses from researching the ways an inclusionary exchange of nurse‐patient differences can inform practice and improve patient care (Canales, 2000).

In providing several contextual analyses regarding the consequences of the dominant‐subordinate relationship in the othering process, Mee (2012) has proposed that a nurse's limited self‐knowledge of his/her own habitually exclusionary behaviour is often manifested in a practice that unconsciously avoids and isolates those patients characterized as problematic or “aggressive”(p. 17). In consequence, this seemingly unconscious practice of exclusion/isolation reduces a patient's capacity to inform nurses of his/her health concerns and significantly puts patients at an increased risk for adverse health outcomes. For example, patients with dementia living in nursing care centres are over 30% more likely to be admitted to a hospital with avoidable complications such as urinary infections, pressure ulcers and dehydration (Quality Care Commission, 2013). Bail et al. (2013) show evidence that “these complications have been specifically associated with aspects of nursing work environments”, where limited direct nurse‐patient contact was correlated with a significant increase of a dementia patient's risk for these complications (p. 7).

These examples illustrate not only the barriers by which othering obstructs knowledge development and puts patients at risk, but also the ways this exclusionary social practice reduces the conceptual space to develop alternative processes and solutions. Indeed, this reduced conceptual space is evidenced by the findings of this analysis, which indicate limited information regarding theoretical propositions and possible interventional solutions for othering.

### 4.2 Conclusion and implications for further research

#### 4.2.1 Summary of findings

Findings from this concept analysis reveal that othering is a seemingly sequential social process with a resolute trajectory aimed at exclusion and marginalization. In addition, evidence suggests that othering occurs as the product of causal and interdependent relationships between key antecedents and attributes that are essential to generating the process. Hence, the othering process begins with an established set of social and psychological conditions that generate a dominant social standard. This dominance is defined and reinforced by creating difference with a subordinate, “the other”. This differentiation is in turn reinforced through exclusion, thereby furthering the dominant‐subordinate relationship in a cyclical or self‐reinforcing manner that promotes marginalization. Generally, othering is gender, ethnically and/or racially based, although this is not exclusively so and can be extended to include other non‐conforming or “problematic” individuals, colleagues and patients. Broad evidence confirms that the effects of the othering process are profound and far‐reaching in nursing practice and across the profession. As a result, othering negatively affects patient care as well as inter‐professional (e.g. nurse‐physician) and intra‐professional (e.g. nurse‐nurse) relationships.

#### 4.2.2 Further research

In a summarizing statement regarding the means by which the nursing profession could possibly evolve to meet the needs of diverse populations Anderson (2004) has suggested that exclusionary social practices could potentially be obviated by fomenting a culture‐wide “paradigmatic shift” toward inclusionary thinking (p. 14). Even in light of Canales’ (2010) unique formulations for disengaging social exclusion through Inclusionary othering, questions remain regarding the means by which a “paradigmatic shift” toward inclusion, as suggested by Anderson (2004), is to be effectuated in nursing practice. Numerous sources have called for broad‐ranging nursing studies investigating the mechanisms by which dominant social standards are used to formulate fixed gender and racial categories in nursing, which facilitate othering and related exclusionary processes, such as marginalization (Lynam, Browne, Kirkham, & Anderson, 2007; Meleis & Im, 1999; Tang & Browne, 2008). Indeed, further investigations are needed to establish a knowledge base regarding the operational elements of othering and an accurate articulation of the way “the other” is generated in clinical and educational settings. Future research might also include investigations into the role of the nursing profession itself and how nursing practice situations affect the pre‐existing conditions conducive to othering (i.e. the antecedents). In addition, researchers could consider exploring and empirically testing theory‐based interventions aimed at disrupting the attributes or essential contributors to othering, such as differentiation and exclusion.

#### 4.2.3 Alternatives to othering

Ultimately, the generative purpose of our concept analysis was to first offer a conceptual basis for understanding how the othering process becomes operable in nursing and then work to investigate the practical and functional measures the nursing profession can take to disrupt exclusionary social processes and their negative consequences. For this reason and in consideration for what our analytical findings have revealed, we believe it is worth emphasizing the potential of alternative inclusionary practices that have already been formulated and put into action as countermeasures to the exclusionary outcomes normally associated with othering. Specifically, our analysis has referenced Canales’ (2010) Inclusionary othering, along with the related concepts of role‐taking and world‐travelling, as potential guides for thinking about alternative approaches to nursing practice and collegial engagement based on inclusionary strategies designed to disrupt the potentially exclusionary outcomes of the othering process (Burbank & Martins, 2010; Lugones, 1987; Phillips & Peterson, 2005).

##### Outsider within

In making a broader multi‐disciplinary survey of constructs that work to disable and/or disrupt the othering process, we find that the work of social theorist/African American scholar Patricia Hill Collins (1986) stands out as exemplary in this regard. Guided by Black feminist literature sources, Collins has formulated a uniquely holistic approach for theorizing the many ways othering may offer a complexity of outcomes. Herein, Collins (1986) has developed an explanatory “outsider within” model illustrating how marginalized individuals use their dominantly assigned role of the excluded “objectified other” to construct self‐identity, self‐valuation and a uniquely informed, albeit “outsider's” perspective of the dominant culture (p. 18). Armed with a keen sense of self, coupled with a unique perspective on how the dominant culture has subjugated them, these “outsider within”/marginalized individuals, “may reveal aspects of reality obscured by more orthodox approaches” (Collins, 1986, p. 15).

In granting these uniquely informed marginalized individuals the descriptive title of “outsider within”, Collins (1986) demonstrates how the distinctive status of an outsider endows and empowers an individual with an informed outside‐to‐inside perspective of the functional social aspects inherent to and “within” the dominant culture. In this way, this specially informed “outsider within” worldview simultaneously explains the status and experience of “the other”, while shedding light on the motives of the dominant force that has relegated “the other” to the margins of society and culture. As described, Collins’ (1986) unique reformulation of “the other” into the “outsider within” has the potential to inform a broad range of research approaches about the methods by which complex exclusionary social processes can be examined from multiple perspectives and how positive and effective responses could be designed to disrupt the negative effects of exclusion.

It is clearly evident that conceptual strategies such as Collins’ (1986) “outsider within”, along with Canales’ (2000) “inclusionary othering”, have much to inform the nursing profession regarding the negative trajectory of the othering process and the possible ways exclusionary practices can be disengaged, while simultaneously empowering inclusionary strategies. As social constructs specifically designed to be antithetical to exclusion, the “outsider within” and “inclusionary othering” formulations help guide us toward ways to re‐think and re‐formulate conceptualizations for new approaches to nursing practice and collegial engagement, which emphasize social processes that support inclusion, while de‐emphasizing and disengaging those processes, such as othering, that generate interpersonal exclusion. And indeed, in Canales’ (2000) and Collins’ (1986) conceptual frameworks, the trajectory of the othering process is not only disrupted, it is made antithetical to itself and disempowered. Herein, we see the potential for alternatives to the othering process in the form of nursing interventions, which are designed to empower the “outsider within” characteristics of patients and nursing colleagues in the interest of creating inclusionary social outcomes.

This work offers a conceptual foundation for considering how othering emerges in the nursing context. Hence, this study is limited in that empirical evidence of othering in nursing practice is neither measured nor addressed. In addition, the authors understand that the experience of being engaged in the phenomenon of othering is highly dependent on context and has a range of epistemological and ontological consequences for the individuals and groups involved. Unfortunately, an accounting for this range of consequences and the implications for the nursing profession are beyond the scope of this study.

In closing we present a brief, yet highly germane excerpt from the writings of literary theorist Edward Said, who over the course of his life and career ardently worked to defend individuals subjected to the process of othering:

It is more rewarding —and more difficult—to think concretely and sympathetically, contrapuntally, about others than only about “us”. But this also means not trying to rule others, not trying classify them or put them in hierarchies… (Said, 1993, p. 336).

Hence, in considering Said's insights and applying them to the nursing profession, it becomes evident that the necessary task for nursing is to develop knowledge to support an inclusionary practice, where the many interpersonal differences that emerge amongst diverse individuals are used to strengthen relationships and improve patient care.

## Conflicts of interest

No conflict of interest has been declared by the authors.
